# Degree, duration, and causes of visual impairment in eyes affected with ocular tuberculosis

**DOI:** 10.1186/1869-5760-4-3

**Published:** 2014-02-01

**Authors:** Soumyava Basu, Sirajum Monira, Rohit Ramesh Modi, Nuzhat Choudhury, Neha Mohan, Tapas Ranjan Padhi, Praveen Kumar Balne, Savitri Sharma, Satya Ranjan Panigrahi

**Affiliations:** 1 LV Prasad Eye Institute, Patia, Bhubaneswar, Odisha 751 024, India; 2 Department of Ophthalmology, Bangabandhu Sheikh Mujib Medical University, Dhaka 1000, Bangladesh; 3 LV Prasad Eye Institute, Hyderabad, Andhra Pradesh 500034, India; 4 District Tuberculosis Center, Capital Hospital, Bhubaneswar, Odisha 751003, India

**Keywords:** Ocular tuberculosis, Visual impairment, Antitubercular therapy

## Abstract

**Background:**

Ocular tuberculosis (TB) can affect nearly every ocular tissue, leading to a variety of vision-threatening clinical manifestations. The goal of this study is to estimate the degree, duration, and causes of visual impairment in eyes affected by ocular TB.

**Results:**

This was a retrospective study of patients diagnosed as ocular TB based on polymerase chain reaction (PCR) for *Mycobacterium tuberculosis* complex. We applied the World Health Organization definition of visual impairment (VI) to affected eye(s), instead of better-seeing eye. Best-corrected visual acuity (BCVA) of <6/18 and ≥6/60 in the affected eye was classified as moderate VI and <6/60 and ≥3/60 as severe VI. Data collected included presenting and final BCVA of affected eyes and the worst BCVA during the study period. Sixty-one eyes of 40 patients were analyzed. Twenty-five patients (52.1%) had bilateral disease. The mean worst BCVA and mean final BCVA (logMAR) were 1.26 ± 0.87 and 0.61 ± 0.85, respectively, and their difference was highly significant (*p* < 0.0001, Friedman test). The median worst and final BCVA results were 1.30 (range 0.0 to 3.0) and 0.20 (range 0.0 to 3.0), respectively. The mean duration of follow-up was 98.34 ± 81.81 weeks. Moderate and severe VIs were seen in 14 (22.9%) and 12 (19.7%) eyes, respectively, during the course of follow up. Twenty eyes (32.8%) had BCVA of <3/60. Moderate VI or worse was most commonly seen in eyes with multifocal serpiginoid choroiditis (*n* = 6; 100%), retinal vasculitis (*n* = 25; 80.6%), and panuveitis (*n* = 12; 80%). The mean duration of visual loss was 25.2 ± 42.37 weeks (median 6.43 weeks, range 0 to 206.42 weeks). Vitreous hemorrhage, complicated cataract, and macular scarring were the common causes of VI.

**Conclusion:**

Ocular TB can result in prolonged visual impairment, more commonly in patients with posterior uveitis or panuveitis.

## Background

Ocular tuberculosis (TB) is among the common causes of infectious uveitis in endemic countries [1]. It has a wide variety of clinical manifestations affecting nearly every ocular tissue. These include anterior uveitis, intermediate uveitis, retinal vasculitis, infectious multifocal serpiginoid choroiditis (MSC), choroidal tuberculoma, subretinal abscess, scleritis, and optic neuritis [2]. However, the diagnosis of ocular TB is often delayed since it is difficult to differentiate these clinical manifestations from other infectious and noninfectious conditions [3]. Additionally, the paucibacillary nature of ocular TB precludes definitive diagnosis in majority of cases [2,4]. Thus, delay in the initiation of specific antitubercular therapy (ATT) can lead to recurrent or chronic inflammation and thereby long-standing visual impairment in the affected eyes [3].

Recent reports on ocular TB have focused on its histopathological features, clinical manifestations, diagnostic criteria, and role of ancillary tests like tuberculin skin test (TST), interferon gamma release assay (IGRA), and chest radiography [4-7]. However, there is no large study on the extent and causes of visual impairment (VI) in this condition. The present study aims to elucidate this vital yet unexplored aspect of ocular TB. We have attempted to evaluate VI in each eye affected by ocular TB (instead of considering visual acuity of only the better-seeing eye) in order to obtain the complete picture of visual morbidity in this disease.

## Methods

We reviewed medical records of all patients with clinically suspected ocular TB, evaluated at our institute between December 2011 and November 2012. We included patients who either had a definitive diagnosis of ocular TB by polymerase chain reaction (PCR) or showed a positive response to antitubercular therapy, i.e., no clinical recurrence 6 months after initiation of antitubercular therapy. The study was approved by the institutional review board and adhered to the tenets of Declaration of Helsinki. We followed a standard protocol for all ocular TB patients. All patients had a detailed recording of history including exposure to active pulmonary tuberculosis. Clinical evaluation included best-corrected visual acuity (BCVA), intraocular pressure, slit lamp evaluation, and indirect ophthalmoscopy. The clinical manifestations and anterior chamber inflammation were classified on the basis of Standardization of Uveitis Nomenclature (SUN) Working Group recommendations, while vitreous haze was graded by the National Eye Institute system [8,9].

All patients were investigated for complete hemogram, erythrocyte sedimentation rate, chest x-ray, TST, and ELISA for HIV; while specific investigations were ordered to rule out infectious (other than TB) and noninfectious conditions that cause similar clinical manifestations. Patients who were clinically suspected with ocular TB (based on above evaluation) and had any grade of anterior chamber cells were considered for anterior chamber paracentesis for PCR test for *Mycobacterium tuberculosis*. We used a multitarget PCR assay targeting the IS6110, MPB64, and protein B gene targets of *M. tuberculosis*[10]. All patients with clinically suspected ocular TB (including PCR-negative patients) were treated with ATT consisting of isoniazid 5 mg/kg/day, rifampicin 450 mg/day if body weight was ≤50 kg and 600 mg/day if body weight was >50 kg, ethambutol 15 mg/kg/day, and pyrazinamide 25 to 30 mg/kg/day for initial 2 months. Thereafter, rifampicin and isoniazid were used for another 4 months. The duration of ATT was restricted to 6 months based on World Health Organization (WHO) recommendations for extrapulmonary TB [11]. Corticosteroid therapy was administered (generally starting on the same day as ATT) by one or more routes - topical, periocular, intravitreal, oral, or intravenous, depending on the anatomical location or severity of inflammation. Patients were generally followed up at weeks 2, 6, 12, 24, and 36 and in between or thereafter, depending on the response to therapy.

The following information was extracted from the patients' records: age; gender; laterality; clinical diagnosis; investigation results including TST, IGRA, chest radiography, and PCR; ocular complications; BCVA; intraocular pressure; inflammatory status at each visit; treatment details (ATT and corticosteroids); additional procedures; and duration of follow-up. The presenting BCVA, worst BCVA during the study period (in case it was different from presenting BCVA), and final BCVA of each eye were noted. For the purpose of our study, VI was defined by applying WHO guidelines to eyes affected by ocular TB [12]. Thus, BCVA of <6/18 and ≥6/60 in the affected eye was classified as moderate VI and <6/60 and ≥3/60 as severe VI. Eyes with BCVA of <3/60 were classified separately. We did not define legal blindness, since only unilateral VI was analyzed. The duration of visual loss was calculated from the visit with BCVA of <6/18 until it improved to ≥6/18. In case of multiple episodes of inflammation, the duration of visual loss in individual episodes was added up to obtain the total duration. The primary cause of decreased visual acuity at each visit was also noted.

Statistical analysis was done using the InStat statistical software version Win 3.0× (GraphPad Software Inc., CA, USA). The impact of gender and laterality on visual loss was analyzed using Mann–Whitney test. Wilcoxon matched-pairs signed-ranks test was used to analyze the impact of treatment. Friedman test (nonparametric repeated measures ANOVA) was used to compare presenting, worse, and final visual acuity. Snellen visual acuity was approximated to logMAR values for statistical analysis, wherever required.

## Results

Sixty-one eyes of 40 patients were analyzed. The patients were nearly equally divided between bilateral (*n* = 21; 52.5%) and unilateral (*n* = 19; 47.5%) cases. The mean age of patients was 34.4 ± 12.11 years (range 6 to 60 years). Twenty-seven (67.5%) were males and 13 (32.5%) were female patients. The mean duration of follow-up was 98.34 ± 81.81 weeks.

The distribution of patients among various clinical patterns of ocular TB is given in Table 1. Retinal vasculitis (*n* = 31 eyes) and panuveitis (*n* = 15 eyes) were the two most common patterns of ocular TB in our series. Overall, the mean worst and final BCVA results in our patients were 1.324 ± 0.89 and 0.698 ± 0.91, respectively. The difference between mean final and mean worst BCVA was found to be highly significant (*p* < 0.0001, Friedman test). Fourteen eyes (22.9%) had moderate VI and twelve eyes (19.7%) had severe VI at some point of follow-up. Twenty eyes (32.8%) had BCVA of <3/60 during this period. Table 1 also mentions various grades and duration of VI noted in each clinical pattern. Moderate VI or worse was most commonly seen in eyes with multifocal serpiginoid choroiditis (*n* = 6; 100%), retinal vasculitis (*n* = 25; 80.6%), and panuveitis (*n* = 12; 80%) and least in anterior uveitis (*n* = 1; 20.0%). The mean presenting visual acuity was worst for panuveitis and best for intermediate uveitis. Overall, the mean duration of visual loss was 25.2 ± 42.37 weeks (median 6.43 weeks, range 0 to 206.42 weeks) for all eyes, which is lowest for anterior uveitis (median 0 weeks) and highest for retinal vasculitis (median 13.57 weeks).

**Table 1:** Distribution of patients and visual impairment among various clinical categories of ocular TB

		**Anterior uveitis**	**Intermediate uveitis**	**Retinal vasculitis**	**Multifocal serpiginoid choroiditis**	**Panuveitis**	**Others**	**Total**
Total number of eyes		5	3	31	6	15	1	61
Mean presenting BCVA ± SD		0.62 ± 0.95	0.73 ± 0.49	0.85 ±0.91	0.55 ± 0.65	0.97 ± 0.79	0.4 ± 0.0	0.82 ± 0.82
Median (range)		0.30 (0.0 to 2.30)	0.50 (0.40 to 1.30)	0.60 (0.0 to 3.0)	0.3 (0 to 1.80)	0.90 (0 to 2.30)	0.4 (0.4to 0.4)	0.50 (0.0 to 3.0)
Mean worst BCVA ± SD		0.84 ± 1.21	1.46 ± 1.34	1.36 ± 0.91	1.02 ± 0.54	1.17 ± 0.69	2.5 ± 0.0	1.26 ± 0.87
Median (range)		0.30(0.20 to 3.0)	0.90 (0.50 to 3.0)	1.30 (0 to 3.0)	0.75 (0.5 to 1.8)	1.30 (0.0 to 2.30)	2.5 (2.5 to 2.5)	1.30 (0.0 to 3.0)
Mean final BCVA ± SD		0.72 ± 1.28	1.2 ± 1.56	0.71 ± 0.88	0.1 ± 0.06	0.49 ± 0.63	0.1 ± 0.0	0.61 ± 0.85
Median (range)		0.20 (0.10 to 3.0)	0.30 (0.30 to 3.0)	0.30 (0.30 to 3.0)	0.10 (0.0 to 0.20)	0.20 (0.0 to 2.30)	0.1 (0.1 to 0.1)	0.20 (0 to 3.0)
Number of eyes with moderate visual loss (BCVA <6/18 and ≥6/60)	P	-	-	5	1	3	-	9
	W	-	1	6	3	4	-	14
	F	-	-	6	-	3	-	9
Number of eyes with severe visual loss (BCVA <6/60 and ≥3/60)	P	-	1	4	-	3	-	8
	W	-	-	8	-	4	-	12
	F	-	-	4	-	1	-	5
Number of eyes with BCVA <3/60	P	1	-	7	1	4	-	13
	W	1	1	11	2	4	1	20
	F	1	1	4	-	1	-	7
Mean duration of visual loss (weeks) ± SD		5.91 ± 13.22	0.38 ± -0.66	36.31 ± 53.37	8.42 ± 8.81	21.27 ± 28.69	11.29 ± 0.0	25.2 ± 42.37
Median (range)		0.0 (0 to 29.57)	0.0 (0.0 to 1.142)	13.57 (0 to 206.42)	5.92 (0 to 24.57)	11.14 (0.0 to 86.571)	11.29 (11.29 to 11.29)	6.43 (0 to 206.42)
Mean duration of follow up (weeks) ± SD		65.23 ± 37.13	123.71 ± 170.97	111.06 ± 88.78	59.071 ± 12.63	90.86 ± 73.68	141.42 ± 0.0	98.34 ± 81.81
Median (range)		41(36.71 to 105.86)	25(25 to 321.14)	63.71(12.42 to 301.71)	63.71 (37 to 69.29)	47.14(21 to 216.42)	141.42 (0 to 141.42)	63.71 (12.43 to 321.14)

BCVA, best-corrected visual acuity; SD, standard deviation; P, presenting; W, worst; F, final.

Various ocular complications and the additional procedures required for management of such complications are given in Table 2. Vitreous hemorrhage, complicated cataract (both *n* = 10), and macular scarring (*n* = 9) were the most commonly observed causes of visual loss. Vitreous surgery was the most commonly required additional procedure in our patients. We did not find any influence of age (*p* = 0.24) or gender (*p* = 0.31) on VI caused by ocular TB.

**Table 2:** Complications and their in various clinical categories of ocular tuberculosis

**Cause of visual loss and additional procedures**	**Anterior uveitis (*n*** **= 5)**	**Intermediateuveitis (*n*** **= 6)**	**Retinal vasculitis (*n*** **= 33)**	**Multifocal serpiginoid choroiditis (*n*** **= 11)**	**Panuveitis (*n*** **= 16)**	**Others (*n*** **= 2)**	**Total**
Pupillary membrane	1	-	-	-	-	-	1
Complicated cataract	1	1	2	1	5	-	10
Secondary glaucoma	1	-	-	-	1	-	2
Vitreous hemorrhage	-	-	9	-	-	1	10
Epiretinal membrane	-	-	2	-	-	-	2
Cystoid macular edema	-	-	5	-	1	-	5
Tractional retinal detachment	-	-	2	-	-	-	2
Optic atrophy	-	-	1	-	2	-	3
Macular scarring/inflammation	-	-	3	4	2	-	9
Cataract surgery	-	1	1	-	2	-	4
Glaucoma surgery	1	-	-	-	1	-	2
Vitreous surgery	-	-	9	-	-	-	9
Retinal photocoagulation	-	-	4	-	-	-	4

## Discussion

Our study analyzes the extent, causes, and duration of VI in eyes affected by ocular TB. Since nearly half our patients had unilateral disease, we preferred to study VI in each affected eye instead of the better eye BCVA, thus getting a wider perspective of visual morbidity in ocular TB. Importantly, unilateral VI (moderate to severe) has been shown to have a measurable impact to health-related quality of life, besides subjective visual function and binocular and stereo acuity [13,14].

The mean age of patients in our series was 34.4 ± 12.11 years, which indicates the impact of ocular TB on the productive age group of our population. The clinical presentation in our patients was predominantly posterior uveitis (retinal vasculitis and MSC) or panuveitis, unlike an earlier report on presumed ocular TB, in which nearly half (45.1%) of the patients had anterior or intermediate uveitis [5]. This could have influenced the degree of VI noted in our series. In general, patients with posterior uveitis or panuveitis had lower mean worst visual acuity than those with anterior or intermediate uveitis. Our results match the previous studies on visual loss in uveitis, though they followed different criteria for classification of visual loss [15-17]. Moderate VI or worse (BCVA < 6/18) was also seen more in patients with posterior uveitis or panuveitis than anterior/intermediate uveitis. Overall, 46 of 61 eyes (75.4%) had moderate VI or worse, during the course of follow up.

The most common causes of visual loss in our study were vitreous hemorrhage, complicated cataract, and macular scarring/ inflammation. Vitreous hemorrhage and CME were commonly seen in eyes with retinal vasculitis, macular scarring was mostly associated with MSC, while complicated cataract was mostly seen in panuveitis. The incidence of CME in our study was lower than that reported for uveitis in general, as we had fewer patients with anterior or intermediate uveitis, and fluorescein angiography and optical coherence tomography were not done routinely in all patients [15-17]. Vitreous hemorrhage was treated with retinal photocoagulation or vitreous surgery as required and often resulted in marked improvement in visual acuity. Eyes with MSC also improved markedly after treatment with ATT and corticosteroid therapy. In general, the mean VA across all categories of clinical presentations, improved significantly (*p* < 0.0001), following ATT and various ancillary treatments. Some eyes with complicated cataract had not received cataract surgery until the last follow-up, indicating that there was further scope of improvement in mean final VA. Thus, our study highlights the effect of ocular complications, the visual morbidity caused by uveitis. Importantly, in epidemiological studies on VI, complications such as cataract and glaucoma attributable to uveitis are often classified separately without the causal diagnosis, leading to falsely low values for uveitis blindness/VI [18].

We did not find any effect of age or gender on the degree of visual impairment. A recent study on mycobacterial ocular inflammation associated delay in diagnosis and age older than 50 years with profound visual loss [3]. We could not calculate the duration of disease for most patients, as the time of onset prior to referral was not clearly documented in all patients. Also, the number of patients in each clinical category was not large enough for statistical analysis of their effect on visual impairment, although, as stated earlier, posterior uveitis or panuveitis generally had worse visual acuity. Finally, we could not separately analyze the impact of ATT on visual impairment in ocular TB, since all patients required simultaneous management of ocular inflammation with corticosteroids and of various complications like vitreous hemorrhage, cataract, and glaucoma.

## Conclusions

Ocular TB and its complications cause moderate to severe visual impairment in majority of affected eyes, especially in eyes with posterior uveitis or panuveitis. Appropriate management of this condition can significantly recover the visual loss.

## Competing interests

The authors declare that they have no competing interests.

## Authors’ contributions

SB was involved in the conception of the study and its design; analysis and interpretation of data; and in drafting, revision, and final approval of the manuscript. SM, RRM, NM, and SS were involved in the acquisition, analysis, and interpretation of data, and in the revision and final approval of the manuscript. NC, TRP, and PKB were also involved in data acquisition and in the revision and final approval of the manuscript. SRP was involved in the conception and design of the study as well as in the revision and final approval of the manuscript. All authors read and approved the final manuscript.

## Authors’ information

NC participated in the project during her fellowship at LV Prasad Eye Institute, Bhubaneswar. PKB and SS were also at LV Prasad Eye Institute, Bhubaneswar, during the period of study.
